# Risk factor analysis for nonocclusive mesenteric ischemia following cardiac surgery: A case-control study

**DOI:** 10.1097/MD.0000000000008029

**Published:** 2017-09-15

**Authors:** Ju Yong Lim, Joon Bum Kim, Sung Ho Jung, Suk Jung Choo, Cheol Hyun Chung, Jae Won Lee

**Affiliations:** Departments of Thoracic and Cardiovascular Surgery, Asan Medical Center, University of Ulsan College of Medicine, Seoul, Republic of Korea.

**Keywords:** cardiac surgery, lactate, laparotomy, nonocclusive mesenteric ischemia, vasoactive-inotropic agent

## Abstract

Although rare, postcardiac surgery nonocclusive mesenteric ischemia (NOMI) is a life-threatening condition. Identifying the risk factors for NOMI during immediate postoperative period may help early detection and intervention, which leads to improved clinical outcomes. The objective of this study was to identify the clinical features and risk factors of NOMI for prognosis identification after cardiac surgery, focusing on immediate postoperative parameters.

Among 9445 patients who underwent cardiac surgery over a span of 9 years, 40 NOMI cases (0.4%) requiring surgical interventions were reviewed. Suspected NOMI was diagnosed by sigmoidoscopy or computed tomography. To identify the risk factors, a control group (case: control = 1:3 ratio) was randomly selected and compared using logistic regression models.

NOMI was diagnosed after a mean of 8.1 ± 9.6 days following cardiac surgery. Age (odds ratio: 1.16, 95% confidence interval: 1.08–1.25, *P* < .001), total vasoactive–inotropic score (VIS), and the maximal lactate level at postoperative day 0 (1.003, [1.001–1.005], *P* = .012), (1.23, [1.04–1.44], *P* = .011) were shown as risk factors. NOMI cases showed persistent hyperlactatemia without washout during the first 48 hours (*P* = .04). Thirty-four cases underwent exploratory laparotomy within a median of 10 (2–356) hours after the diagnosis, but only 17 patients (42.5%) survived. Compared with survivors, nonsurvivors showed higher total VIS at diagnosis, higher lactate levels during the first 24 hours postoperatively, and more frequently required extensive bowel resection (*P* < .05).

Old age, postoperative high-dose vasoactive-inotropic use, and persistent high lactate level during the first 24 hours postsurgery were identified as risk factors for NOMI. Lactic acidosis and necrotic-bowel extent at surgical exploration were associated with poor survival.

## 1 Introduction

Nonocclusive mesenteric ischemia (NOMI) was first described in 1958,^[1]^ and is one of the most fatal complications following cardiac surgery. Although its incidence rate is very low, the mortality rate is reported to be as high as 90%.^[2,3]^ As early detection and surgical intervention are considered crucial to outcomes of NOMI,^[4]^ many risk factors such as age, the use of an intra-aortic balloon pump, the use of vasopressors, and increases in inflammatory markers have been previously proposed.^[2,3,5–7]^ However, those clinical settings are neither uncommon nor unique after cardiac surgeries. Moreover, the timing to suspect NOMI in patients with those risk factors remains unclear. For example, in their recent study for risk factor analysis for NOMI,^[5]^ Groesdonk et al^[5]^ suggested important postoperative risk factors such as norepinephrine use >0.1 μg/kg/min and lactate concentrations >5 mmol/L. However, the timing of these findings was not clearly described, so that causal relationship could not be derived. The pathological mechanism of NOMI is poorly understood as well; however, its development after cardiac surgery is closely associated with cardiopulmonary bypass or intraoperative factors^[8,9]^ combined with preoperative factors. As the day of surgery is the most hemodynamically unstable period after cardiac surgery, use of high-dose vasoactive inotropic drugs during this period for any reason including low cardiac output or bleeding may aggravate the mesenteric injury initiated by cardiopulmonary bypass or intraoperative factors. Therefore, immediate postoperative parameters may reflect mesenteric malperfusion or potential risk of NOMI. Therefore, we focused on the immediate postoperative parameters to identify the risk factors of NOMI after cardiac surgery and investigate the prognosis of NOMI.

## 2 Methods

### 2.1 Study population

From January 2007 to October 2015, a total of 9445 consecutive cardiac surgeries were performed at our institution. Among these cases, NOMI requiring surgical consultation for intervention occurred in 40 patients (0.4%). To perform a case-control study, these 40 patients needed to be compared with the control group out of the remaining 9405 patients. Therefore, 120 of 9405 patients were randomly selected from the cardiovascular surgery database at our institution as a control group by using the table of random sampling numbers (case: control = 1: 3). The Institutional Review Board of our institute approved this study (IRB number: 2015–0713).

### 2.2 Diagnosis of NOMI

Patients who were suspected with NOMI underwent urgent abdominal and pelvic computed tomography (CT). A radiologist confirmed the presence of NOMI when ischemic bowels were noticed in the CT with intact distal perfusion of superior and inferior mesenteric arteries without thrombus or occlusion. To assess the extent or severity of ischemic bowels, sigmoidoscopy was performed at bedside.

### 2.3 Modified vasoactive-inotropic score

Postoperative use of vasoactive-inotropic drugs likely affects mesenteric arterial perfusion in addition to intraoperative cardiopulmonary bypass. Comparing the total amount of vasoactive-inotropic drugs used by patients during the immediate postoperative period is difficult because there are several different types of commonly used vasoactive-inotropic drugs, and the infusion dosages are adjusted hourly according to patients’ individual vital signs. Therefore, vasoactive-inotropic score (VIS), which is a method established in pediatric patients and described by Gaies et al,^[10]^ was modified and used to compare the dose and types of inotropes and vasopressors administered postoperatively. VIS was calculated as follows:

VIS per hour = dopamine dose (μg/kg/min) + dobutamine dose (μg/kg/min)

+ 100 × epinephrine dose (μg/kg/min)
+ 100 × norepinephrine dose (μg/kg/min)
+ 10,000 × vasopressin dose (U/kg/min)
+ 10 × milrinone dose (μg/kg/min)

Total VIS (postoperative day 0) = sum of hourly VIS scores during the first 24 hours following surgery for each patient.

Phenylephrine was not included because it was not postoperatively used according to physician's preference.

### 2.4 Statistical analysis

Statistical analyses were performed using SPSS version 21 (IBM Institute, Cary, NC). The data are expressed as mean ± standard deviation or median (interquartile range [IQR]) for continuous variables, and numbers and percentages for categorical variables. Preoperative and postoperative measurements were compared using Student *t* test. The *χ*^*2*^ test or Fisher exact test was used to compare the categorical variables and to assess the statistical significance between the 2 groups. *P* value ≤.05 was considered to be statistically significant for all comparisons.

Regarding the risk factor analysis for NOMI, variables with *P* values of <.1 were first considered for logistic regression models. Next, we performed bootstrapping to select variables that were identified as independent risk factors of the case in at least 60% of the bootstrap samples out of 1000 times with backward stepdown option.

Follow-up period for each patient was calculated from the date of the operation to the date of death or last contact.

## 3 Results

**Figure 1:**
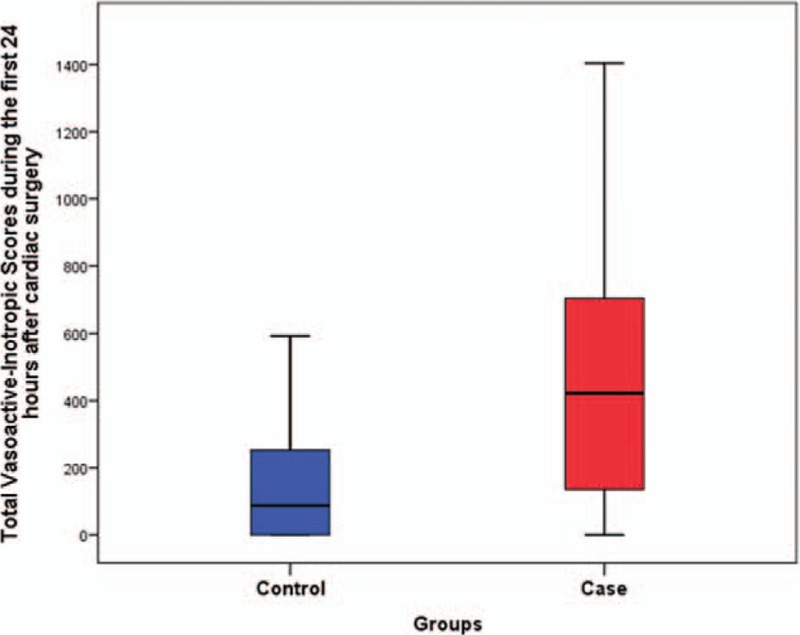
Total vasoactive-inotropic scores during the first 24 h after cardiac surgery.

**Figure 2:**
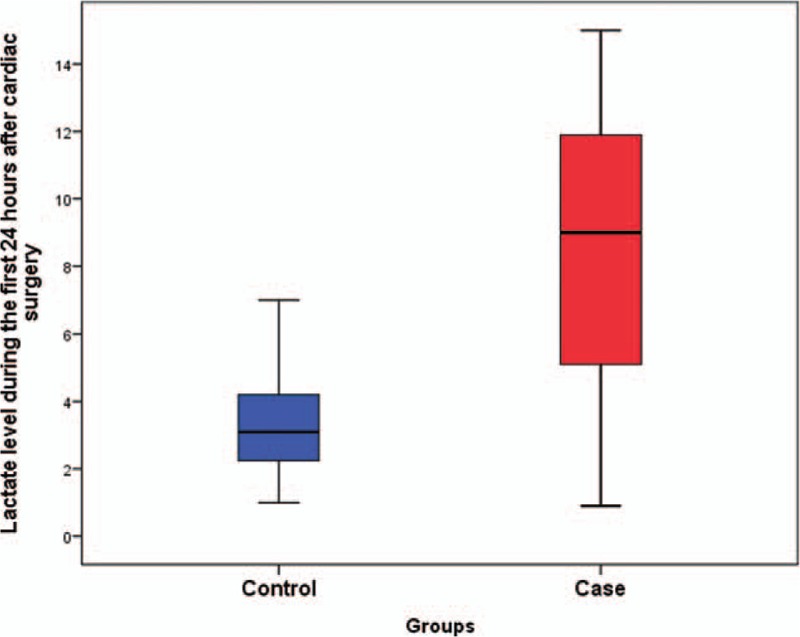
Maximal lactate level during the first 24 h after cardiac surgery.

The baseline characteristics between NOMI and the controls are summarized in Tables 1 and 2. In the case group, the mean age was significantly higher, and more female patients were included. Intraoperatively, the cardiopulmonary bypass (CPB) time, or the aortic cross-clamp time, was significantly longer in the case group (*P* < .05). An isolated valve operation was performed in 17 patients (42.5%), and isolated aortic surgery was performed in 13 patients (32.5%). Four patients underwent a combined valve surgery with an aorta replacement, and 1 patient underwent combined coronary artery bypass grafting (CABG) with aortic surgery. Isolated CABG was performed in 4 patients, and combined valve with CABG was conducted for 1 patient. Consequently, aortic surgery was significantly higher in the case group (*P* < .001), whereas CABG was significantly lower. A total of 30% of the patients in the case group received extracorporeal membrane oxygenator (ECMO) support postoperatively, a rate that was significantly different from that of the control group (*P* < .001). The rate of re-exploration for bleeding was significantly higher in the case group. In addition, the total VIS and maximal lactate level during the first 24 hours following surgery were significantly higher in the case group (*P* < .001) (Figs. 1 and 2).

**Table 1:**
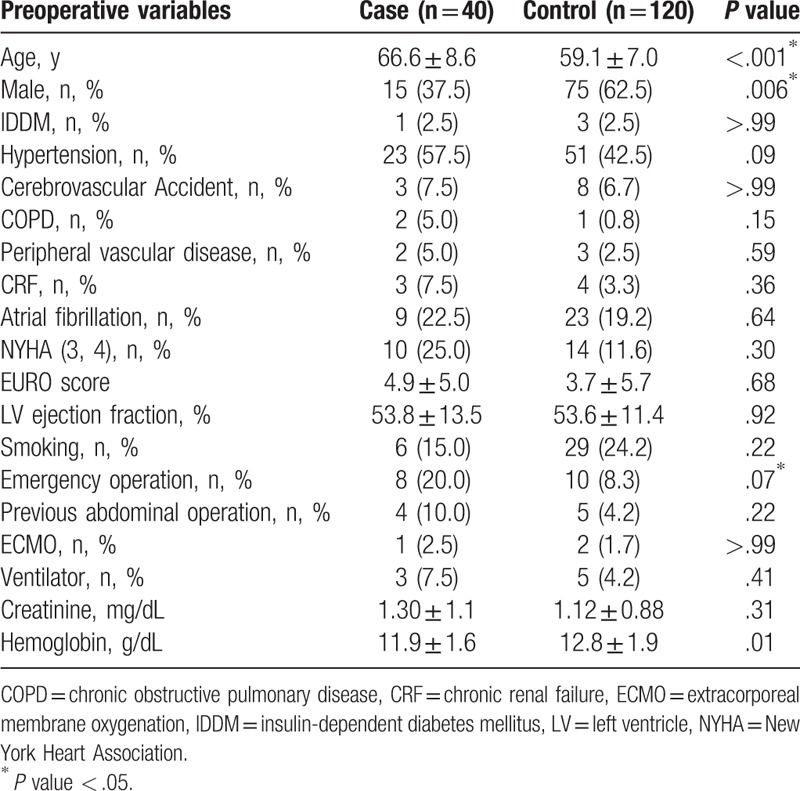
Preoperative characteristics of the patients.

**Table 2:**
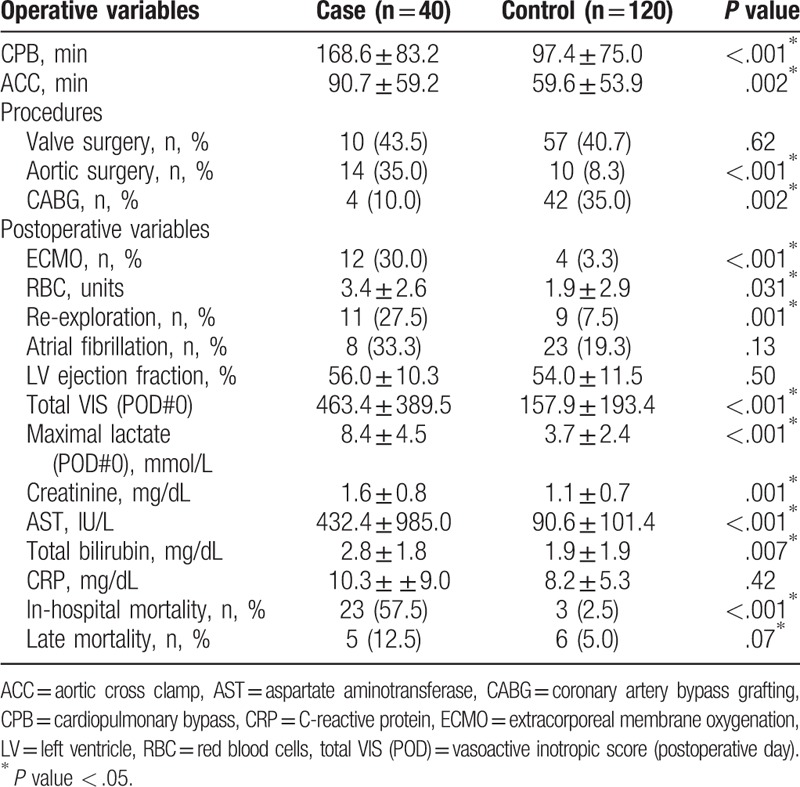
Operative and postoperative patient data.

### 3.1 Univariate and multivariate analyses for risk factors of NOMI after cardiac surgery

In the univariate analysis, age, female, emergency operation, CPB time, aortic surgery, postoperative ECMO support, re-exploration, and postoperative total VIS as well as the maximal lactate during the first 24 hours following surgery were found to be significant risk factors for NOMI. Age, sex, emergency operation, CPB, aortic surgery, CABG, postoperative ECMO, re-exploration, total VIS (POD#0), maximal lactate, creatinine, total bilirubin, and aspartate aminotransferase (AST) were included in the final multivariate analysis model. The multivariate analysis demonstrated that age (odds ratio (OR): 1.2, 95% confidence interval (CI): 1.1–1.3, *P* < .001), an increase in the postoperative total VIS during the first 24 hours by 100 scores (OR: 1.3, 95% CI: 1.1–1.6, *P* = .012), and the postoperative maximal lactate level during the first 24 hours (OR: 1.2, 95% CI: 1.0–1.4, *P* = .011) increased the risk for NOMI following heart surgery. These findings are summarized in Table 3.

**Table 3:**
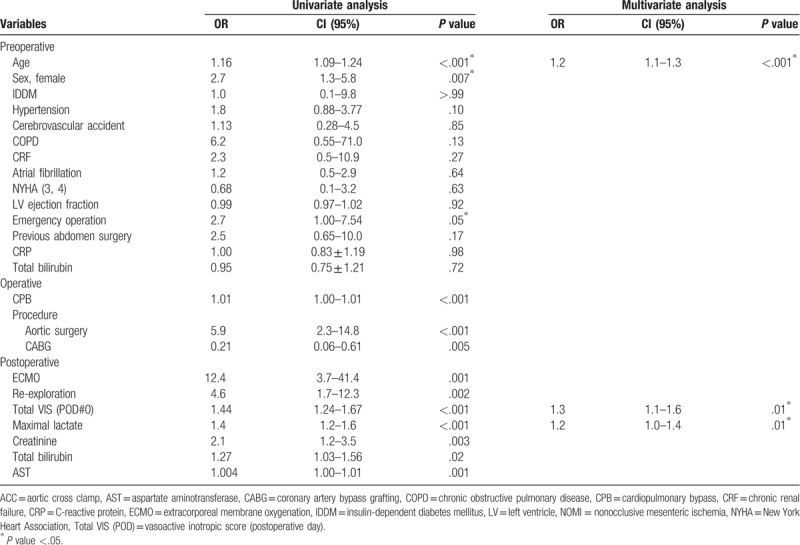
Univariate and multivariate analyses for NOMI following cardiac surgery.

### 3.2 Survival of patients diagnosed with NOMI

Forty patients were diagnosed with NOMI after a mean of 8.1 ± 9.6 days following cardiac surgery. Among these patients, 6 patients did not receive exploration due to their severely deteriorated general condition or refusal by a legal guardian. The remaining 34 patients underwent emergency abdominal exploration for resection. A total of 10 (25%) and 13 (32.5%) patients underwent small-bowel and large-bowel resections, respectively. Both the small-bowel and large-bowel resections were needed in 10 patients (25%). In 1 patient, the necrotic bowel was regarded as nonresectable because the necrosis was too extensive. Emergency surgical interventions were carried out at a median interval of 9.5 hours (2–356 hours) after the diagnosis of NOMI, with 17 early survivors (42.5%). During the mean follow-up period of 21.4 ± 7.7 months, late mortality occurred in 5 patients, which resulted in freedom from overall mortality rate of 27.5% at 1 year.

As presented in Table 4, the survivors and nonsurvivors did not show any significant differences in terms of preoperative or intraoperative variables. Postoperatively, more patients underwent ECMO support and received high-dose vasoactive-inotropes during the first 24 hours in the nonsurvivor group (*P* < .05). Maximal lactate level during the first 24 hours as well as at the time of diagnosis of NOMI was higher in the nonsurvivors (*P* < .05). The extent of bowel resection showed a negative correlation with survival (*P* = .035).

**Table 4:**
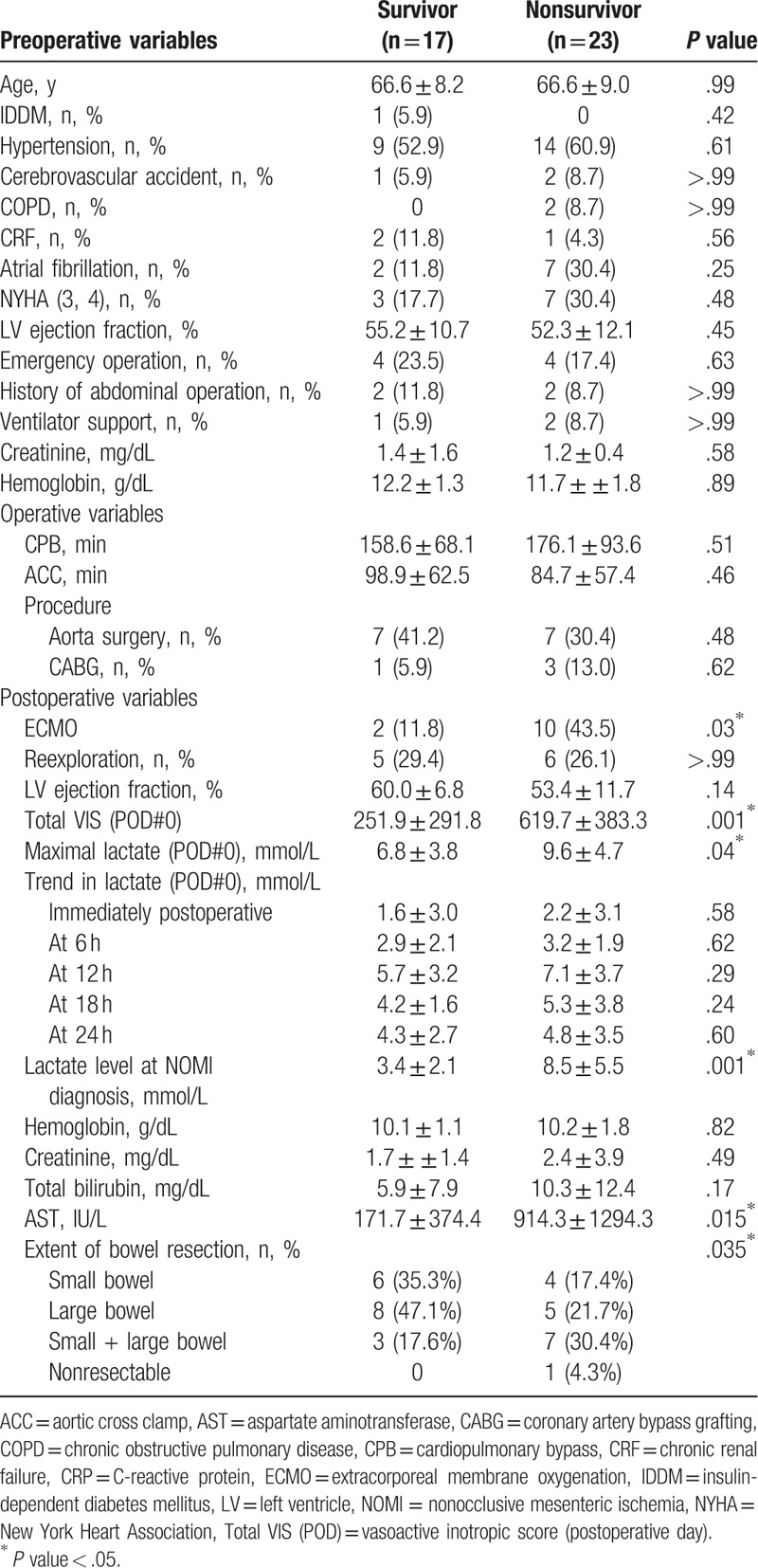
Comparison between survivors and nonsurvivors with NOMI.

### 3.3 Postoperative lactate trends between case and control group

**Figure 3:**
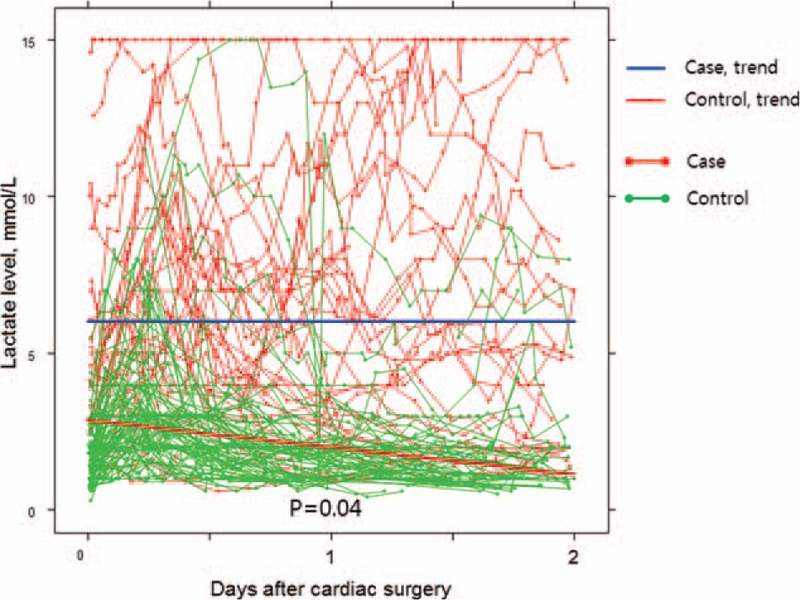
Lactate trend during the first 48 h after cardiac surgery.

As the maximal lactate level during the first 24 hours was identified as a significant risk factor for NOMI, we performed the random coefficients model to identify whether the rate of change in the lactate level differed between groups. In this analysis, the rate of change in lactate level was significantly different between groups (*P* = .0007). In control group, estimated lactate change per day was −0.28 with a standard error of 0.04 (*P* < .001), whereas estimated change was 0.54 with a standard error 0.37 in NOMI cases (*P* = .15). These lactate trends were shown to be more prominent during the first 48 hours after surgery, in which estimated interval change in control group was −0.86 (*P* < .001), as opposed to the NOMI patients who showed persistent hyperlactatemia without any significant change (*P* = .99) (Fig. 3). Regarding survival, estimated lactate level in nonsurvivors was 1.97 times higher than that of the survivors, but the trends in each group did not show any statistical difference.

## 4 Discussion

NOMI is a rare but fatal complication following cardiac surgery, and its pathophysiology is poorly understood. Microcirculatory alterations in the mesenteric artery during or after a cardiopulmonary bypass contribute to intestinal hypoperfusion. This impaired blood flow may lead to compromised intestinal mucosal integrity, which aggravates inflammatory response and bacteremia.^[8,9,11–13]^ Moreover, it is assumed that microcirculatory changes are consequences of vasopressor use during or after cardiac surgery.^[14]^ Regardless of the understanding of the NOMI pathological mechanisms, the most important factor is to detect patients at risk of NOMI as early as possible. Mesenteric artery angiography is considered the gold standard diagnostic tool for NOMI, because it may also be used as a therapeutic tool by infusing vasodilator (e.g., papaverine) directly into the mesenteric artery.^[3]^ However, angiography is invasive and rarely feasible in severely ill, hemodynamically unstable patients. Therefore, despite low sensitivity, less-invasive methods (e.g., contrast abdominal, pelvic CT) are preferred as alternative diagnostic tools in clinical settings.^[4]^ In our study, NOMI was diagnosed via contrast CT, and sigmoidoscopy was performed to confirm bowel ischemia and necrosis. Regardless of the diagnostic tools, it is important that surgical exploration not be delayed to obtain diagnostic imaging if the patient shows peritoneal signs or if NOMI is highly suspected, because intestinal resection is the mainstay of treatment.^[15]^

Although the early identification of NOMI is crucial, suspicion of NOMI remains challenging because the clinical signs are not specific. Oliguria, hypotension, and lactic acidosis are not rare in the immediate postoperative period following cardiac surgery. In addition, symptoms such as abdominal pain tend to be underestimated under analgesic/sedative administration.^[16,17]^ Nevertheless, we aimed to identify the risk factors of NOMI by comparing NOMI patients to the control group who were randomly selected from the non-NOMI patients. As summarized in Table 1, demographics including EuroSCORE were similar between groups except for age and sex. The only identified risk factor before surgery was age. Also, we considered several intraoperative and postoperative factors for our study. Specifically, we thoroughly examined postoperative laboratory values during the first 24 hours after cardiac surgery, because NOMI can occur as a consequence of intraoperative CPB and perioperative vasopressor administration. A multivariate analysis revealed old age as well as high total VIS and lactate levels on postoperative day 0 as risk factors for NOMI. These results are quite similar to those from other retrospective and prospective studies.^[5,18]^ The unique aspect of total VIS is that it considers the dose of specific inotropes and vasopressors but also their duration of use. Although continuous norepinephrine infusion was administrated in the immediate postoperative period, total VIS might be low if the duration of administration was short; thus, the patient is less likely to develop NOMI. The total VIS cutoff value during the first 24 hours for predicting NOMI was 400 (sensitivity 53%, specificity 88%). Regarding the maximal lactate level during the first 24 hours, it is well known that lactic acidosis following cardiac surgery is associated with poor clinical outcome.^[19,20]^ In the same context, if lactic acidosis persists regardless of stable vital signs and adequate postoperative cardiac output, NOMI may be suspected as the source of lactic acidosis. The best predictive value of the maximal lactate level was >5 mmol/L (sensitivity 75%, specificity 86%), which coincided with the result from the study by Groesdonk et al.^[5]^ Also, the absolute value of the peak lactate level after cardiac surgery might not be as important as lactate trend,^[21]^ because there are other factors that may be associated with hyperlactatemia in the immediate postoperative period. The important thing is that if the persistent hyperlactatemia exists despite optimal postoperative management with volume resuscitation and necessary medical support, physician should suspect or consider ongoing organ malperfusion. One of the suspects of malperfused organs should be the instestines. Lactate trends found in our study also showed that persistent hyperlactatemia existed in the case group, whereas lactate was gradually washed out in the control group.

Once NOMI is diagnosed, a poor clinical course usually ensues. Despite emergency surgical exploration and the resection of the necrotic bowel, the mortality rate is very high. Allen et al reported a mortality rate of 66.7%, which was similar to our results at 70%. The laboratory findings (e.g., lactate and AST) were worse in the nonsurvivors, which might be the end-product of progressive multiorgan failure resulting from necrotic bowels. Furthermore, no survivors underwent more extensive necrotic bowel resection, which might imply earlier detection when bowel ischemia was localized in either the small bowel or the large bowel. This may improve survival even though surgical intervention is required.

### 4.1 Study limitations

There are several limitations in our study. First, this is a retrospective observational study and not a randomized controlled study. Therefore, the comparison between the 2 groups may be biased with potential confounding factors. However, we utilized case-control design to decrease any potential bias. Second, the incidence of NOMI may be underdiagnosed because patients had extremely poor general condition when they were diagnosed with NOMI, and thus were not able to undergo any more diagnostic imaging tests. Third, the application of VIS, which was established in pediatric patients after cardiopulmonary bypass, has not yet been validated in adults. However, it seems to be a feasible method for comparing the total amount of vasoactive-inotropic drugs during the first 24 hours after surgery, because there is currently no standardized method for comparing the effects of vasoactive drugs in adult patients.

In conclusion, we identified old age, high dose of and prolonged use of vasopressors, and lactic acidosis during the first 24 hours following cardiac surgery as risk factors of NOMI. In addition to the high lactate level, persistent hyperlactatemia was found in NOMI cases during the first 48 hours. Signs of organ failure and the extent of the necrotic bowel at the time of surgical exploration were associated with poor survival in NOMI cases.
